# Acute Physiological Stress Promotes Clustering of Synaptic Markers and Alters Spine Morphology in the Hippocampus

**DOI:** 10.1371/journal.pone.0079077

**Published:** 2013-10-24

**Authors:** Veronica Sebastian, Jim Brian Estil, Daniel Chen, Lisa M. Schrott, Peter A. Serrano

**Affiliations:** 1 Department of Psychology, Hunter College, New York, New York, United States of America; 2 Department of Pharmacology, Toxicology and Neuroscience, Louisiana State University Health Sciences Center, Shreveport, Louisiana, United States of America; 3 The Graduate Center of CUNY, New York, New York, United States of America; SUNY Downstate Medical Center, United States of America

## Abstract

GluA2-containing AMPA receptors and their association with protein kinase M zeta (PKMζ) and post-synaptic density-95 (PSD-95) are important for learning, memory and synaptic plasticity processes. Here we investigated these synaptic markers in the context of an acute 1h platform stress, which can disrupt spatial memory retrieval for a short-term memory on the object placement task and long-term memory retrieval on a well-learned radial arm maze task. Acute stress increased serum corticosterone and elevated the expression of synaptic PKMζ while decreasing synaptic GluA2. Using co-immunoprecipitation, we found that this stressor promotes the clustering of GluA2, PKMζ and PSD-95, which is consistent with effects reported from overexpression of PKMζ in cell culture. Because PKMζ overexpression has also been shown to induce spine maturation in culture, we examined how stress impacts synaptic markers within changing spines across various hippocampal subfields. To achieve this, we employed a new technique combining Golgi staining and immmunohistochemistry to perform 3D reconstruction of tertiary dendrites, which can be analyzed for differences in spine types and the colocalization of synaptic markers within these spines. In CA1, stress increased the densities of long-thin and mushroom spines and the colocalization of GluA2/PSD-95 within these spines. Conversely, in CA3, stress decreased the densities of filopodia and stubby spines, with a concomitant reduction in the colocalization of GluA2/PSD-95 within these spines. In the outer molecular layer (OML) of the dentate gyrus (DG), stress increased both stubby and long-thin spines, together with greater GluA2/PSD-95 colocalization. These data reflect the rapid effects of stress on inducing morphological changes within specific hippocampal subfields, highlighting a potential mechanism by which stress can modulate memory consolidation and retrieval.

## Introduction

The ability of stress paradoxically either to enhance or impair memory consolidation and retrieval is a well-documented phenomenon [1]. In particular, the hippocampus, an area widely known for its role in learning and memory processing, is vulnerable to stress-induced neuroendocrine responses affecting structure and function [2]. The degree to which the hippocampus is affected by stress depends upon the timing and type of stressor [1,3]. The effects of stress in rodent models are contingent on various parameters, including stressor duration and intensity, ranging from mild to severe [4]. Typically mild stressors induce enhanced performance for spatial and fear conditioning tasks [5], while severe stressors produce impairments in memory function irrespective of whether the stress is acute or chronic [6]. These effects are associated in part with changes in hippocampal neuronal structure and spine density. Chronic and/or severe stressors induce rapid changes in spine density in CA1 [7] while promoting dendritic retraction in CA3 [8]. Stress-induced spine changes in CA3 coincide with deficits in hippocampal function involving radial arm maze, Y-maze, and water maze performance [9–11]. The mechanisms by which stress induces these changes in structure and function of the hippocampus are largely unknown.

In the adult brain, axons and dendrites remain relatively stable, while dendritic spines appear to be the primary site of structural plasticity [12]. Spines form the post-synaptic component of excitatory synapses and are capable of rapid development, expansion, contraction and elimination [13–15]. Typically, spines are characterized by their morphology, based on a dynamic continuum. The relationship between the diameter of the spine head and length of the neck provides an indication of spine development. Spines develop from filopodia, characterized by thin, long dendritic protrusions, lacking a head or post-synaptic density. Stubby spines usually show major hallmarks of synapses, including post-synaptic densities, but lack necks. In contrast, long-thin and mushroom spines have distinct necks and wider heads [16]. Large spines generally persist for weeks to months and form strong synapses. In contrast, small spines are generally transient, forming weaker synapses [13,15,17]. Based on these properties, mushroom-type spines have been hypothesized to represent physical substrates of long-term memories, i.e., memory spines, while small or stubby spines represent the capacity for adaptive, experience-dependent rewiring of neuronal circuits, i.e., learning spines [17,18].

Recent findings have also identified a potential mechanism for clustering of synaptic markers known to play a role in the development of excitatory synapses [19]. These protein clusters involve protein kinase M zeta (PKMζ) and the α-amino-3-hydroxy-5-methyl-4-isoxazolepropionic acid receptor (AMPAR) subunit GluA2, together with the post-synaptic density protein 95 (PSD-95). PKMζ is a persistently active kinase that is necessary for maintaining the late-phase of long-term potentiation (LTP) [20,21] and increasing EPSCs by selectively upregulating the AMPAR insertion [22,23]. PKMζ activity is important for various forms of long-term memory involving spatial appetitive and avoidance memories, conditioned reflex memory, and taste avoidance memory [24–27]. Recent studies have confirmed that the insertion of GluA2 subunits into the synapse is a key function of PKMζ activity necessary for long-term memory maintenance [23,28]. In cultured cells, a chemical LTP paradigm increased clustering of PKMζ/PSD-95 while PKMζ overexpression increased spine colocalization of GluA2/PSD-95 [19]. Similarly, PKMζ overexpression has also been shown to increase mature spine levels without affecting overall spine density [29]. 

Though new studies are beginning to elucidate the mechanisms and functionality of synaptic protein clusters in memory, it remains unclear how these clusters of colocalizing proteins are impacted by stress, a common and dynamic modulator of hippocampal function. Furthermore, the functional aspects of these changes are largely unknown. Thus, we address the following questions: How are clusters of key synaptic markers affected by an acute physiological stress? How are dendritic spines affected by this stress across hippocampus subfields? Finally, how are these synaptic protein clusters participating in stress-induced changes in spine morphology? 

## Materials and Methods

### Subjects / Stress Treatment

Young adult (9-15 weeks) male Sprague-Dawley rats (Charles River; Boston, MA) were pair-housed in plastic cages (48 x 27 x 16 cm) containing hardwood bedding. Animal quarters were maintained at constant temperature (22±1°C) and relative humidity (40-50%) with a 12h light/dark cycle (lights on at 8AM). Food (Harlan Teklad; Frederick, MD) and water were available *ad libitum*. Rats were subjected to an acute stressor by being placed on a small, elevated platform (pedestal = 1.22 m high; platform = 12.7 x 12.7 cm) for 1 hour. All procedures were performed in accordance with the NIH Guide for the Care and Use of Laboratory Animals and approved by the Institutional Animal Care and Use Committee at Hunter College.

### Object Placement Task

**Figure 1:**
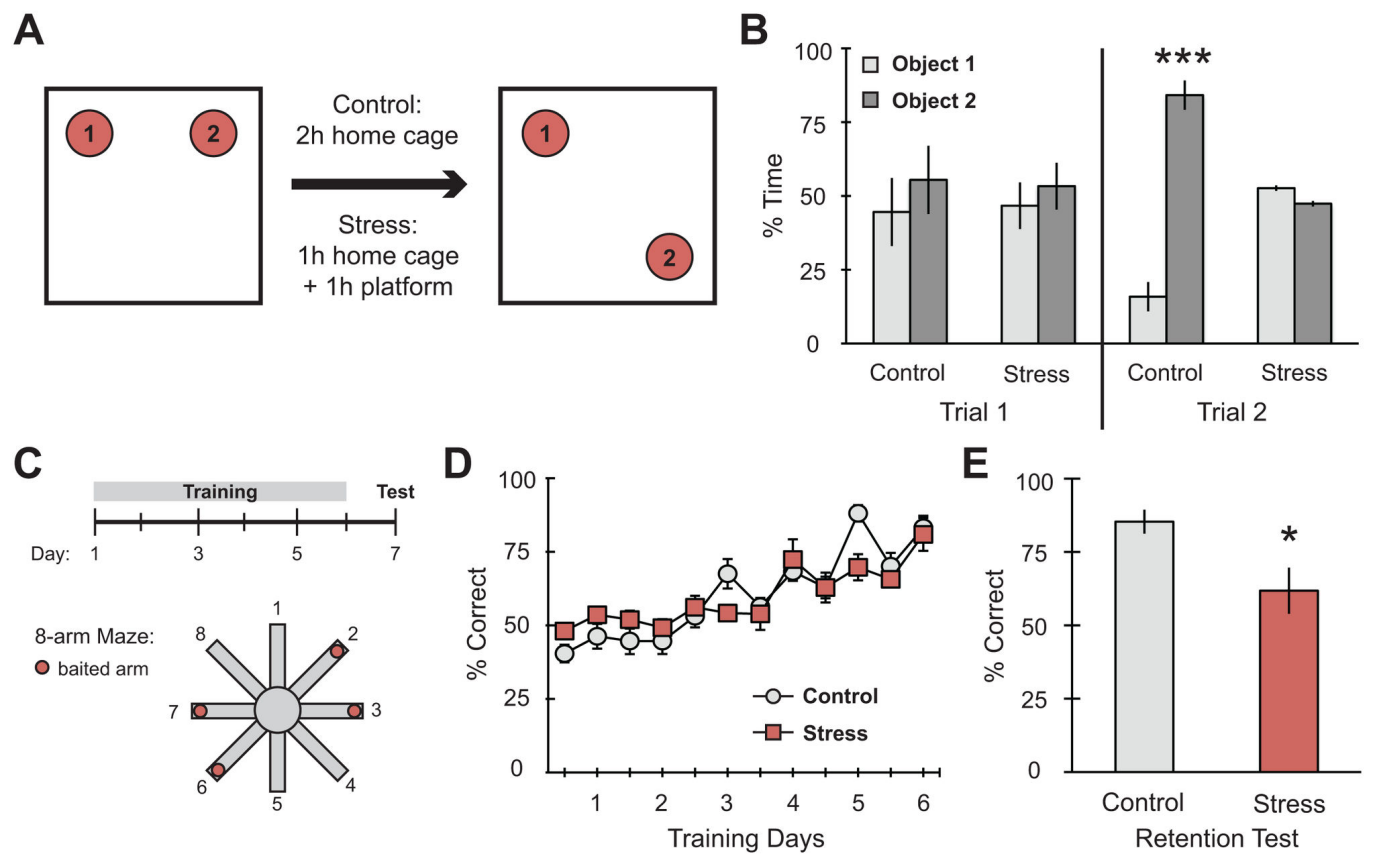
Spatial memory on the object placement and radial arm maze tasks is impaired after platform stress. (A) Schematic diagram of the object placement task experimental design. (B) There were no significant differences in time spent exploring objects during Trial 1 of the object placement task prior to stress. Controls showed a significant increase in exploration of the object in the novel location (object 2) while stress subjects failed to make a dissociation between objects (n = 6 control, 7 stress). (C) Schematic diagram of the radial arm maze task experimental design. (D) Rats learned the radial arm maze equivalently and significantly improved their performance over training days prior to stress (n = 5 control, 5 stress). (E) Stress prior to the retention test impaired memory retrieval 24h after the last training trial. For all graphs, *p<0.05, ***p<0.001.

Rats received a 5min habituation to the empty open field (0.91 x 0.91 meters) 30 min prior to the task. They were then placed into the field with two identical objects. The time spent exploring each object was recorded. Following the first trial subjects were returned to their home cages for one hour. Stressed rats were then placed on the platform for 1h while naïve controls remained in their home cage. Two hours after trial 1 and immediately post-stress, all subjects were given trial 2, in which one of the objects (Object 2) was moved to a novel location (Figure 1A). If the animal spent more time exploring the object in the novel location, it was considered to have demonstrated intact memory.

### Radial Arm Maze

Procedures are as previously published [26,30]. Briefly, rats were food-deprived to 85% of their initial body weight. Before training all rats were habituated to the maze and to the sweetened oatmeal mash, which served as the food reward. During training each rat received 10 consecutive trials per day over a period of 6 days (60 trials total). In each trial the same 4 out of 8 arms had food located at the end of each arm (Figure 1C). The number of arms entered to collect the food reward was recorded. Each trial had a maximum latency of 180s. A percent correct score was calculated for every trial and averaged across trial blocks (5 trials each). Twenty-four hours after the last training trial, 3 additional trials were given as a retention test. A subset of the trained rats were given a 1h platform stress immediately before the retention test to measure the effects of platform stress on spatial memory retrieval. 

### Tissue Collection

**Figure 2:**
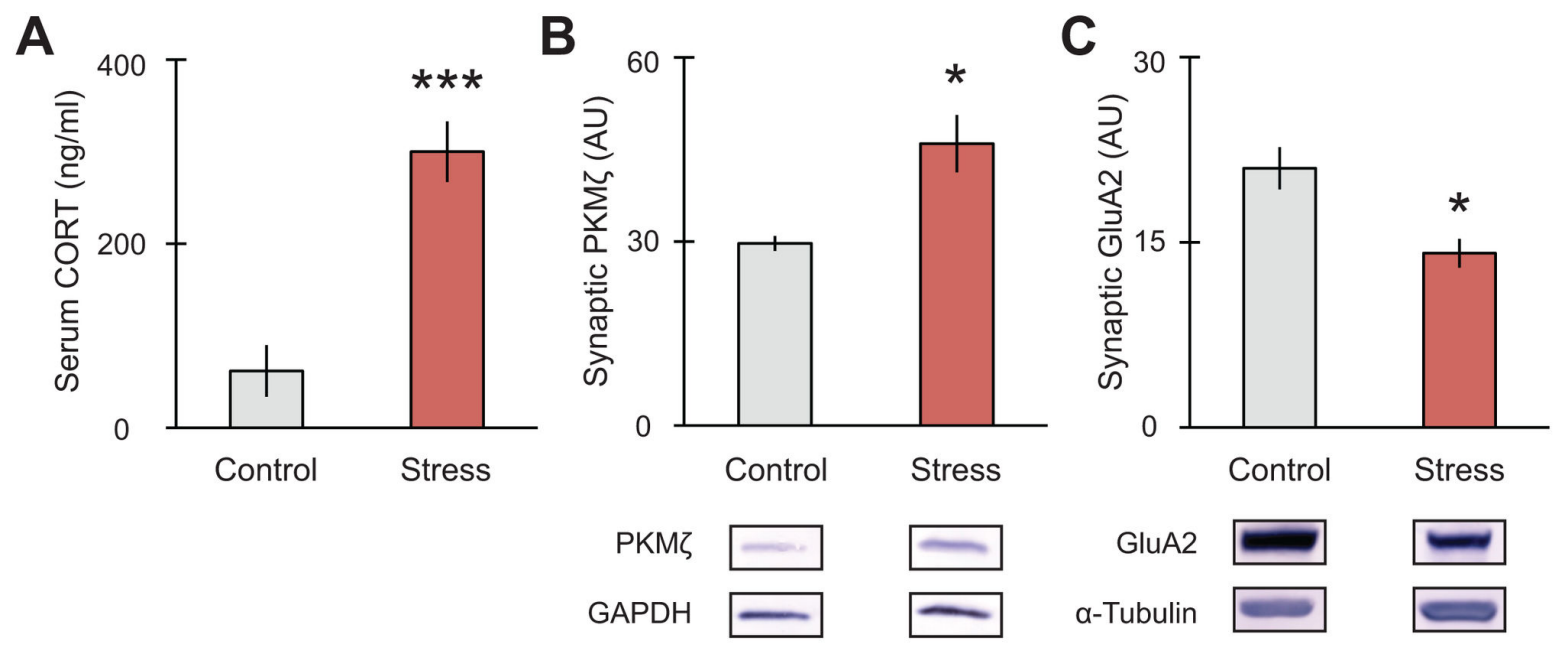
Stress increases serum corticosterone and differentially affects synaptic markers for memory in the hippocampus. (A) Serum corticosterone increased immediately after 1h elevated platform stress (n = 6 control, 10 stress). (B) Synaptic PKMζ in hippocampus increased with platform stress while (C) synaptic GluA2 expression decreased after stress (n = 4 control, 8 stress). (D) Representative blots shown. For all graphs, *p<0.05, ***p<0.001.

**Figure 3:**
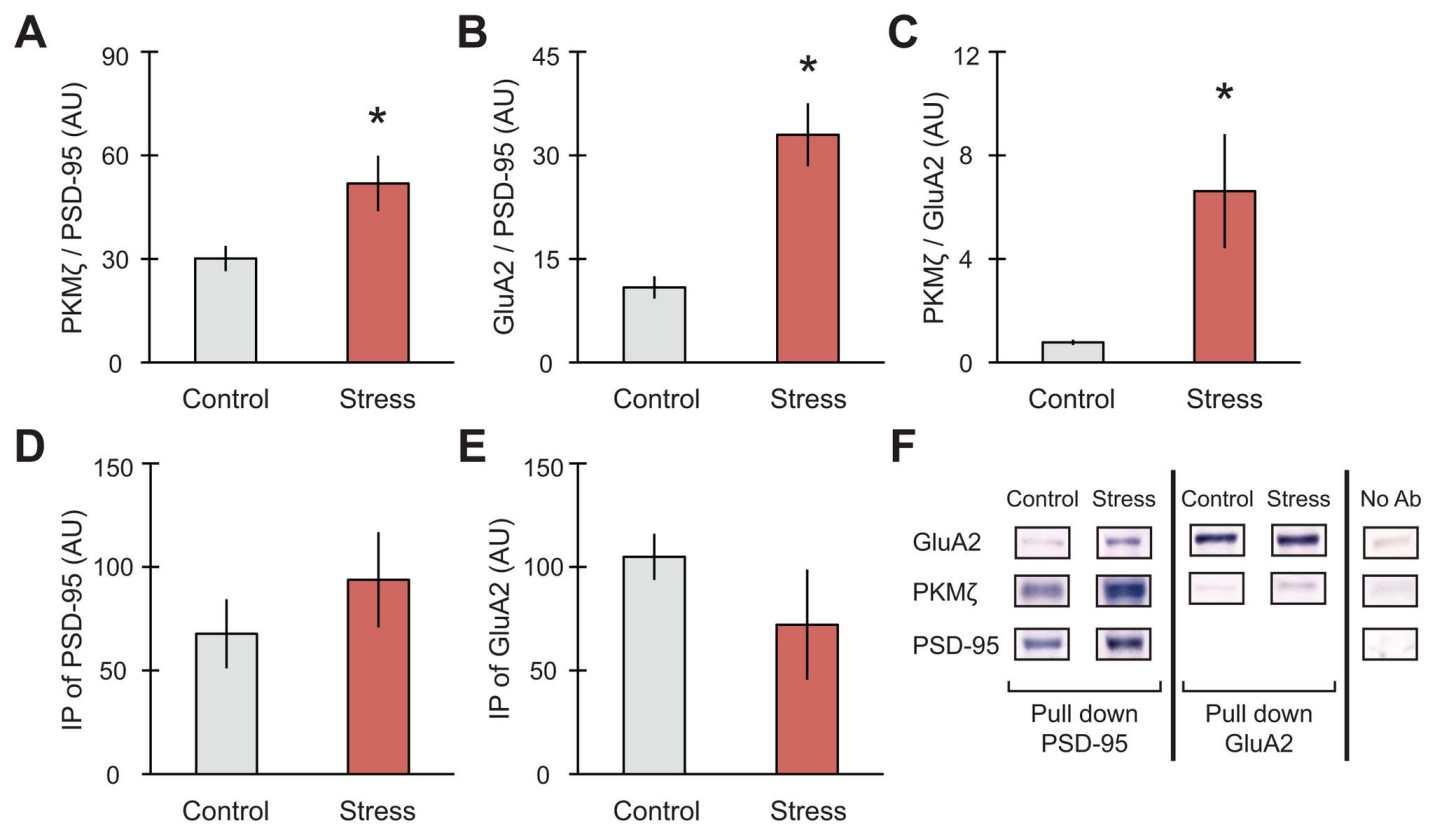
Acute stress increases synaptic clustering of GluA2, PKMζ and PSD-95 in hippocampus. (A) Co-IP of PKMζ with PSD-95 significantly increased (n = 6 control, 8 stress), as did (B) Co-IP of PSD-95 with GluA2 (n = 5 control, 8 stress) and (C) Co-IP of PKMζ with GluA2 (n = 6 control, 6 stress). Overall levels of PSD-95 (D) or GluA2 (E) did not differ between conditions. (F) Representative immunoblots for IP shown. For all graphs, *p<0.05.

**Figure 4:**
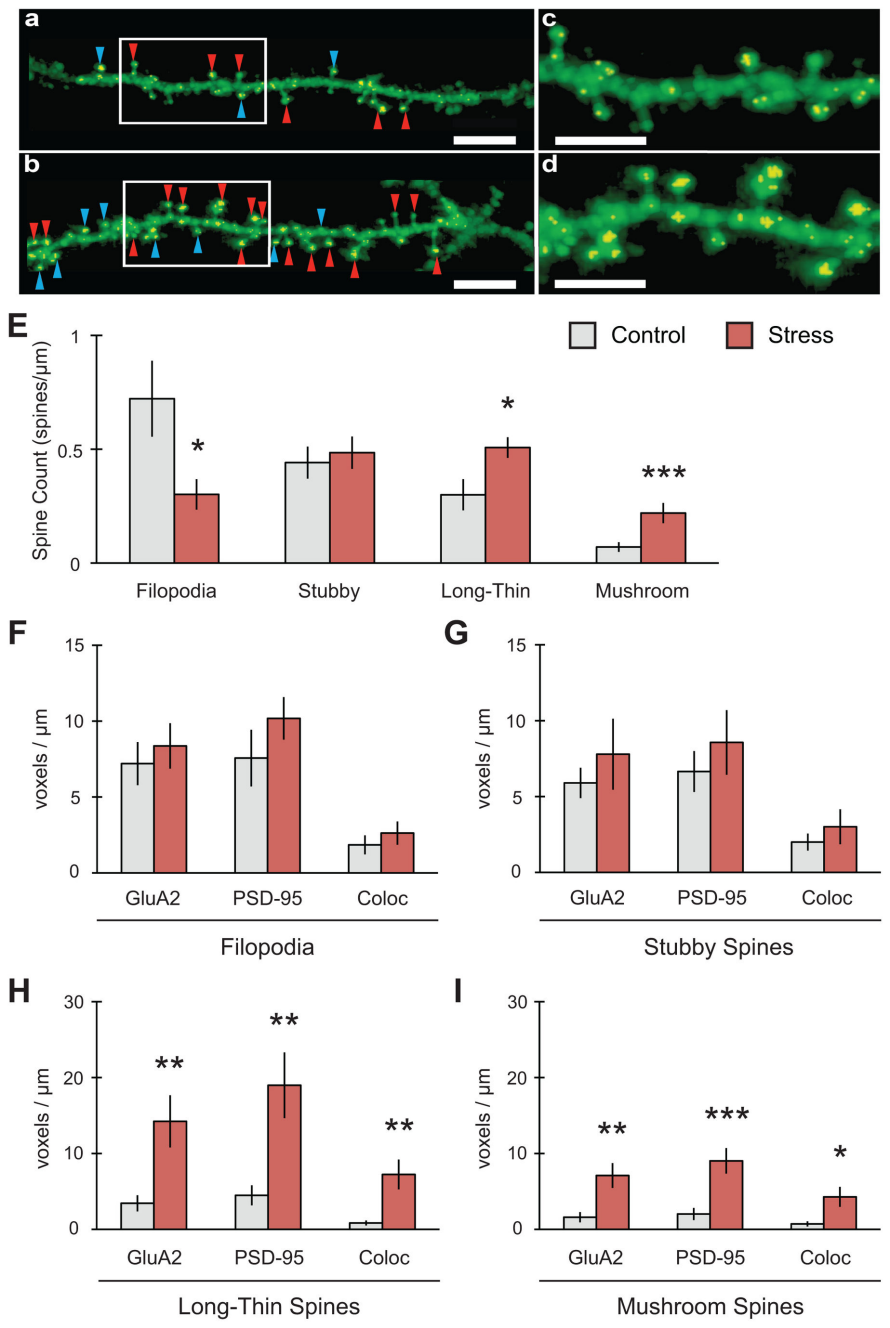
Stress increases both mature spines and colocalization of GluA2 with PSD-95 in area CA1. (A-D) Representative 2D reconstruction of dendrites for control (A, C) and stress (B, D) conditions (scale bar = 5mm for A-B; 3mm for C-D). Golgi-Cox indicated in green, colocalization of synaptic markers in yellow. Red arrowheads indicate long-thin spines, blue arrowheads indicate mushroom spines. (E) Stress increased long-thin (n = 10 control dendrites, 12 stress) and mushroom (n = 11 control, 12 stress) spine counts with a concomitant decrease in filopodia (n = 10 control, 11 stress) and no change in stubby spines (n = 11 control, 12 stress). (F-G) No changes in GluA2, PSD-95 or their colocalization were found in either filopodia (n = 10 control, 8 stress) or stubby spines (n = 12 control, 12 stress). (H) Long-thin spines showed increases in GluA2, PSD-95 and their colocalization (n = 10 control, 10 stress). (I) Mushroom spines showed increases in GluA2, PSD-95 and in their colocalization (n = 11 control, 9 stress). For all graphs, *p<0.05, **p<0.01, ***p<0.001.

**Figure 5:**
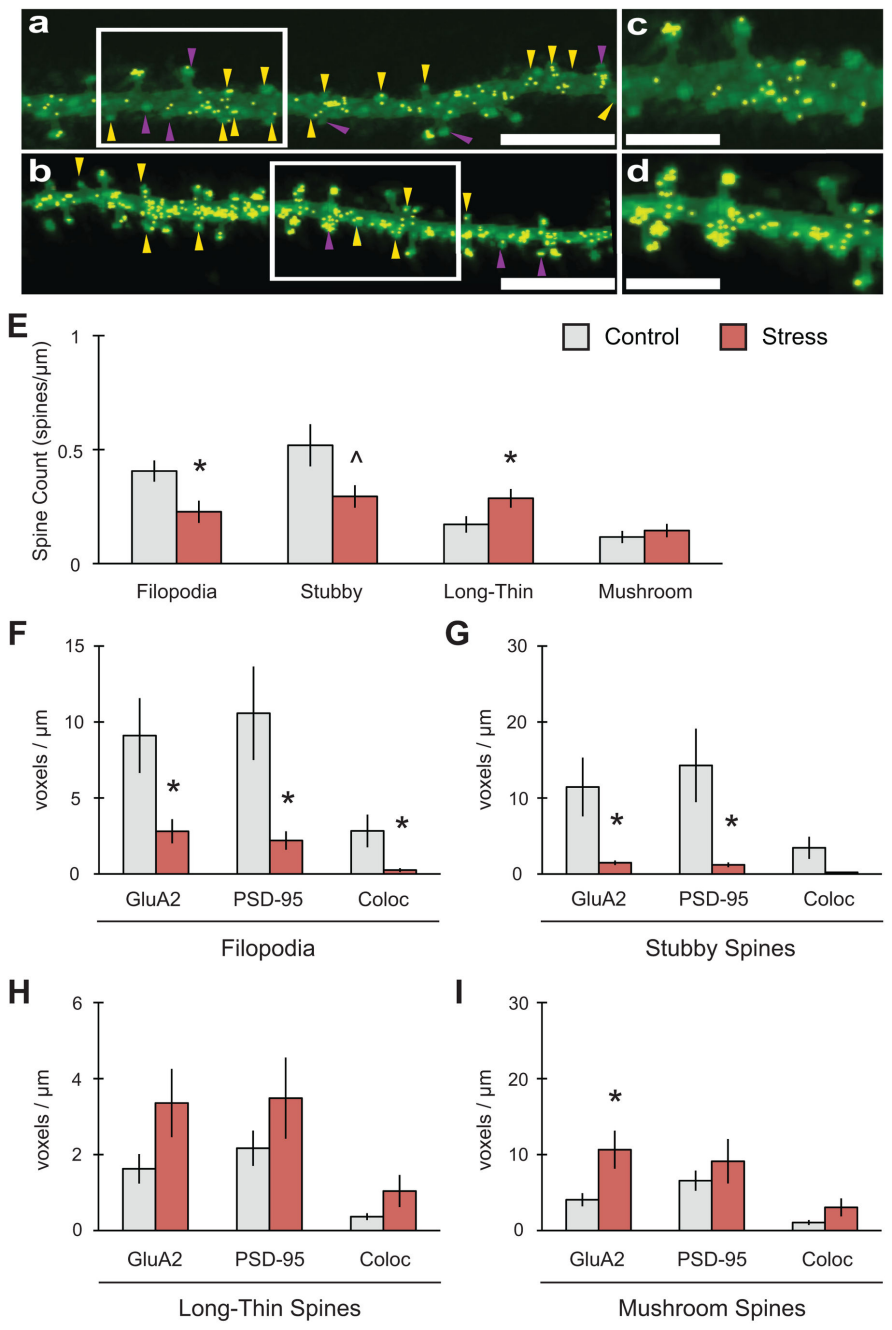
Stress reduces both immature spines and colocalization of GluA2 with PSD-95 in area CA3. (A-D) Representative 2D reconstruction of dendrites for control (A, C) and stress (B, D) conditions (scale bar = 5mm for A-B; 3mm for C-D). Golgi-Cox indicated in green, colocalization of synaptic markers in yellow. Yellow arrowheads indicate stubby spines, purple arrowheads indicate filopodia. (E) Stress decreased filopodia (n = 11 control dendrites, 9 stress) with a concomitant increase in long-thin spines (n = 11 control, 11 stress). Stubby spines (n = 12 control, 11 stress) also demonstrated a trend towards decreased expression, while mushroom spines (n = 12 control, 11 stress) showed no change overall. (F) Filopodia showed a decrease in GluA2, PSD-95 and in their colocalization (n = 9 control, 8 stress). (G) Stubby spines showed a decrease in GluA2 and PSD-95 but no significant change in their colocalization (n = 9 control, 8 stress). (H) No changes in GluA2, PSD-95 or their colocalization were found in long-thin spines (n = 11 control, 11 stress). (I) Stress increased GluA2 expression in mushroom spines but had no effect on PSD-95 or colocalization (n = 12 control, 9 stress). For all graphs, ^p=0.05, *p<0.05.

**Figure 6:**
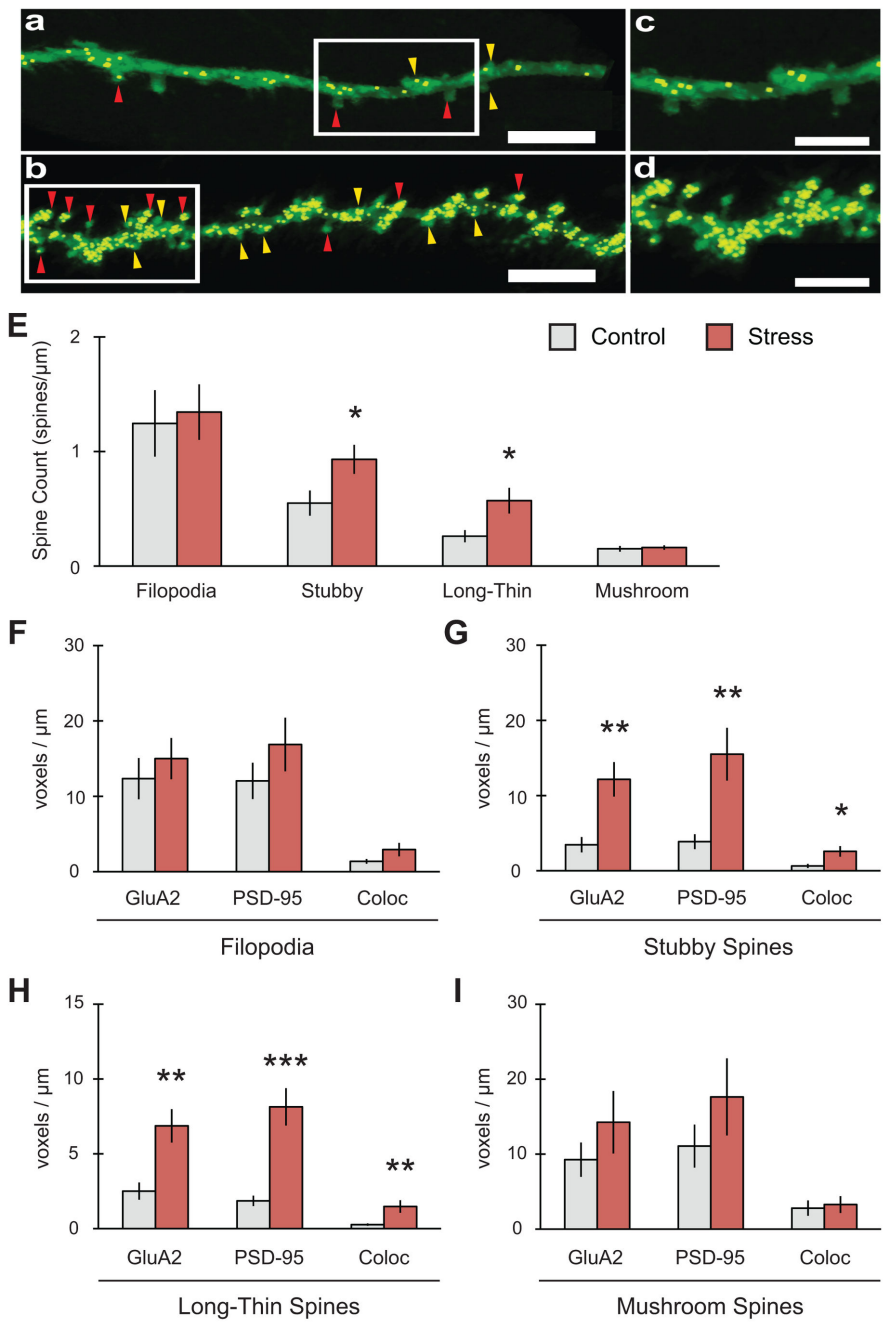
Stress selectively increases both mature and immature spine types along with colocalization of GluA2 with PSD-95 in the outer molecular layer of the dentate gyrus. (A-D) Representative 2D reconstruction of dendrites for control (A, C) and stress (B, D) conditions (scale bar = 5mm for A-B; 3mm for C-D). Golgi-Cox indicated in green, colocalization of synaptic markers in yellow. Yellow arrowheads indicate stubby spines, red arrowheads indicate long-thin spines. (E) Stress increased stubby (n = 11 control dendrites, 11 stress) and long-thin (n = 12 control, 10 stress) spine counts. (F) No changes in GluA2, PSD-95 or their colocalization were observed in filopodia (n = 11 control, 10 stress). (G) Stubby spines showed increases in GluA2, PSD-95 and their colocalization (n = 10 control, 10 stress). (H) Long-thin spines showed increases in GluA2, PSD-95 and their colocalization (n = 12 control, 12 stress). (I) No changes were observed in mushroom spines (n = 12 control, 12 stress). For all graphs, *p<0.05, **p<0.01, ***p<0.001.

All tissue samples were collected immediately following the 1h platform stress or retention test. For experiments in Figures 2-3, subjects were rapidly decapitated, trunk blood was obtained and brains were removed for hippocampal dissections. Blood samples were spun at low speed (3,000g for 10min at 4°C) to obtain sera for corticosterone analysis. Following ether extraction of the sera, corticosterone was analyzed by Enzyme-linked immunosorbent assay (ELISA) kit (Neogen; Lexington, KY). Plates were read in a BioPlex Bead Array Reader (BioRad; Hercules, CA). Whole hippocampi were stored at -80°C until processed for fractionation or co-immunoprecipitation (Co-IP). For experiments in Figures 4, 5, 6, subjects were deeply anesthetized with pentobarbital and perfused with 4% paraformaldehyde to prepare the tissue for Golgi-Immunohistochemistry.

### Fractionation

Fresh frozen whole hippocampi were homogenized in TEE buffer containing protease and phosphatase inhibitors and spun at low speed (3,000g for 5min at 4°C) to remove the nuclear pellet. Samples were then ultracentrifuged (100,000g for 30min at 4°C) to separate out the cytosolic fraction in the supernatant [31]. The remaining pellet was resuspended in homogenizing buffer containing 0.001% Triton X-100 and incubated on ice for 1h and then spun in the ultracentrifuge (100,000g for 1h at 4°C). The pellet from this spin is the synaptic fraction [32]. Fractions were prepared for immunoblotting by standardizing total protein concentrations using a BCA assay (Pierce; Rockford, IL). 

### Co-Immunoprecipitation

This technique used the Dynabeads Co-Immunoprecipitation kit as per manufacturer’s instructions (Life Technologies; Grand Island, NY). Briefly, magnetic epoxy beads were coupled with antibody overnight, washed, and incubated with whole hippocampal homogenate for one hour. Eluted samples were denatured and analyzed by immunoblotting. Relative differences between the protein pulled down and the protein that co-immunoprecipitated with it indicate the amount of interaction in vivo as a function of the experimental manipulations. 

### Immunoblotting

Samples were subjected to SDS-PAGE and transferred to nitrocellulose membranes. The membranes were incubated overnight at 4°C with primary antibodies selective for: PKMζ (1:1000, Santa Cruz Biotechnology; Santa Cruz, CA), GluR2 and PSD-95 (1:1000, EMD Millipore; Billerica, MA). After incubation with appropriate alkaline-phosphatase conjugated secondary antibodies, the reaction product was visualized using BCIP/NBT (KPL; Gaithersburg, MD). GAPDH or α-Tubulin (1:2000, EMD Millipore; Billerica, MA) was used as a loading control. The membranes were scanned and band density measured using ImageJ (NIH; Bethesda, MD).

### Golgi-Immunohistochemistry

Following perfusion, brains were post-fixed overnight. Whole brains were then rinsed with 0.4M Sorensonon’s phosphate buffer before being placed in Golgi-Cox solution containing 5% potassium chromate, 5% potassium dichromate and 5% mercuric chloride for 2d. Samples were moved to fresh Golgi-Cox solution for 14d before cryoprotection in 30% sucrose solution for 2-3d. Whole brains were then snap frozen in isopentane on dry ice and cut serially into 100 µm coronal sections, containing septal hippocampus. The Golgi-Cox stain was then developed: slices were washed for 1min in deionized water, incubated for 30min in 50% NH_4_OH, incubated for 30min in fixer solution (Kodak; Rochester, NY), washed for 1min in deionized water and stored in phosphate buffer at 4°C until immunostaining. Sections were incubated in 0.05M glycine in 0.2% Triton X-100 in PBS for 30min to quench peroxidase activity and washed in PBS before being incubated in blocker containing 5% NGS, 5% BSA and 0.5% Triton X-100 in PBS overnight at 4°C. Sections were then incubated in primary antibodies selective for GluR2 and PSD-95 (1:1000 in PBS, EMD Millipore; Billerica, MA) for 48h at 4°C and washed in PBS before being incubated in secondary antibodies (1:1000 in PBS, Life Technologies; Grand Island, NY) for 2h at room temperature. Sections were washed in PBS, mounted onto slides and coverslipped with ProLong Gold antifade reagent (Life Technologies; Grand Island, NY). Fluorescent-labeled secondary antibodies were matched to laser excitation wavelengths (488nm, 568nm) to optimize emission spectra. Furthermore, the metallic deposits in dendritic structures were reflected by a 514nm laser-line to resolve processes through laser scanning confocal microscopy. Sections were paired (one from each condition) and imaged in batches using a Leica SP2 laser scanning confocal microscope (Leica Microsystems; Buffalo Grove, IL). Images were taken in a 1024x1024 format, at 12-bits, to achieve 0.146 voxels per micron. Each scan line was averaged twice. During the resolution of the Golgi-Cox stain and synaptic markers, minimum gain settings were used. Confocal images were taken of CA1 tertiary dendrites projecting into stratum radiatum, CA3 tertiary dendrites, and tertiary dendrites from the outer molecular layer (OML) of the dentate gyrus (DG). Z-stacks (4-6 µm; Z-step size 0.041 µm for CA1; 0.122 µm for CA3 and OML) were acquired using preset laser power and gain settings. IMARIS 7.5 (Andor Technology; Belfast, Northern Ireland) was used to reconstruct z-stacks into 3D models for analysis. Models were constructed with optimized presets as described by Spiga et al. [33]. Additional presets were used to construct models of synaptic markers, which were colocalized with respect to dendritic structures. Using customized settings based on spine parameters as previously described [34,35], IMARIS Filament Tracer module was used to detect, quantify, and characterize spine structures (Table 1). Multiple tertiary dendrites, approximately 15-75 µm, were analyzed per subject (n = 3 subjects per condition, total 8-12 dendrites per condition). For the colocalization of synaptic markers for each spine type, masked binary channels (inside voxels set to 150) were created for the dendritic shaft, filament, and each spine class. Colocalized voxels in the dendritic shaft alone were subtracted from the total amount of colocalized voxels in each filament to determine the number of colocalized voxels within spines. The number of colocalized voxels for individual synaptic markers was also determined by colocalizing the masked channel for each marker with the masked filament channel of interest. 

**Table 1:** Classification of spine morphology using IMARIS software.

**Type**	**Parameters**
Long-Thin spines	Mean_width(neck)*2 < length(spine) AND mean_width(neck) <= max_width(head)
Mushroom spines	Mean_width(head) > mean_width(neck)
Stubby spines	Length(spine) < 1
Filopodia	Mean_width(head) <= mean_width(neck)

### Statistical Analyses

For experiments in Figure 1, performance on the object placement task was analyzed using a two-way ANOVA with a Bonferroni post-hoc. Training on the radial arm maze was analyzed using a two-way, repeated measures ANOVA. Performance on the radial arm maze retention test was analyzed with an unequal variance, two-tailed t-test. For experiments in Figures 2-3, corticosterone and immunoblotting data were also analyzed using unequal variance, two-tailed t-tests. For experiments in Figures 4, 5, 6, we followed the recommendations of a recent guide to statistical techniques applicable to repeated-measures data [36]. We accounted for the biological and statistical non-independence of data obtained from multiple dendrites within the same subjects by applying a mixed model in JMP 10.0 software (SAS Institute; Cary, NC) to avoid pseudoreplication. The Restricted Maximum Likelihood (REML) method allows for regression modeling of repeated-measures and requires no assumptions regarding the distribution of the data. Experimental groups were treated as a fixed effect while multiple dendrites were treated as a random effect nested within each subject.

## Results

### Acute platform stress disrupted spatial memory retrieval

Previous studies have shown that acute stressors, such as novelty exposure and footshocks, induce impairments in spatial memory retrieval [37–39]. Accordingly, we began by evaluating the behavioral effects of our stress paradigm (1 hour on an elevated, unstable platform) on two spatial memory tasks, object placement and radial arm maze. In the object placement task, subjects explored two identical objects in an open arena (Figure 1A). After a 2h delay, subjects were placed back in the arena for trial 2, in which one of the objects was moved to a novel location. Typically, rats spend more time exploring the object in the new location, demonstrating an intact memory of the original positioning of the objects. Indeed, control animals showed a significant increase in exploration of the object in the novel location (object 2). Stressed subjects, given 1h platform stress immediately before retention testing, failed to make a distinction between objects (Figure 1B; Object F_(1,22)_ = 90.62, ***p = 0.0001; no stress effect; Interaction F_(1,22)_ = 123.36, ***p = 0.0001; Bonferroni post-hoc: Control Obj. 1 vs Obj. 2 ***p = 0.001, Stress Obj. 1 vs Obj. 2 not significant; n = 6 control, 7 stress). These data suggest that 1h platform stress significantly disrupts performance on a spatial memory task.

However, because the object placement task involves only two trials over the course of a few hours, it is difficult to definitively determine whether the stressor disrupted memory retrieval or affected consolidation. Therefore, we examined the effects of platform stress on memory retrieval using the radial arm maze task, which allows for consolidation to take place over a period of days prior to retrieval (Figure 1C). Rats were given 10 trials per day for 6 consecutive days, as previously reported [26,30]. Prior to stress, both groups of rats learned equivalently and significantly improved performance over training days (Figure 1D; Time F_(11,88)_ = 26.28, ***p = 0.0001; no stress effect; n = 5 control, 5 stress). Twenty-four hours after the last training trial, rats in the stress condition were given 1h platform stress immediately before the retention test, resulting in impaired retrieval (Figure 1E; t _(6)_ = 2.658, *p = 0.038). Specifically, stressed subjects made more reference memory errors compared to controls (mean ± SEM: control = 0.667 ± 0.236, stress = 1.667 ± 0.279; t _(6)_ = 2.739, *p = 0.029; figure not shown) but showed no deficit in working memory (mean ± SEM: control = 0.467 ± 0.309, stress = 0.600 ± 0.194; t _(6)_ = 0.365, p = 0.728; figure not shown). Together with the results of the object placement task, these data indicate that our acute stress paradigm can significantly alter memory retrieval for either a short- or long-term spatial memory. 

### Platform stress increased sera corticosterone and modulated synaptic expression of long-term memory markers

Next, we examined the physiological and neurochemical changes induced by the platform stress paradigm. To confirm the effects of platform stress on hypothalamic-pituitary axis (HPA) activation and to determine its impact on the expression of synaptic markers within the hippocampus, groups of rats were given either 1h exposure on the elevated platform or 1h in their home cage prior to tissue collection. Figure 2A shows that serum corticosterone from trunk blood increased after 1h on the elevated platform (t _(13)_ = 5.510, ***p = 0.001; n = 6 control, 10 stress), indicating a robust physiological stress response. Additionally, we analyzed select synaptic markers associated with long-term memory. We focused on PKMζ, a brain-specific molecule known to play a key role in long-term memory maintenance for object placement and radial arm maze [26,40]. We also examined GluA2 expression within the synapse, which is particularly important for maintaining object placement memory [28]. Specifically, the dynamic interaction between PKMζ and GluA2 results in the stabilization of the subunit in the synapse. Loss of this stabilization by PKMζ inhibition leads to internalization of the receptor and memory impairment [28]. In the hippocampus, platform stress significantly increased synaptic PKMζ expression (t _(7)_ = 3.366, *p = 0.012; n = 4 control, 8 stress) with a concomitant decrease in GluA2 (t _(5)_ = 3.330, *p = 0.021; n = 4 control, 8 stress) compared to controls (Figure 2B-C). These data suggest that increasing synaptic PKMζ without increasing GluA2 is an expression pattern associated with stress-induced memory impairment. We hypothesize that increases in PKMζ as a consequence of stress may be altering spine densities, creating a spine density expression pattern that interferes with memory and LTP function. Therefore, to test whether stress promotes synaptic dysfunction, we looked for alterations in synaptic clustering.

### Platform stress increased synaptic clustering

GluA2, PKMζ and PSD-95 are known to create synaptic clusters in culture, which are significantly increased by PKMζ overexpression [19]. In culture, corticosterone also increases GluA2 mobilization [41]. Given that we found stress increased synaptic PKMζ (Figure 2B) and serum corticosterone (Figure 2A), we asked whether platform stress could then affect the expression of GluA2, PKMζ and PSD-95 synaptic clusters. Figure 3 shows significant increases in Co-IP of PKMζ with PSD-95 (Figure 3A; t _(9)_ = 2.462, *p = 0.036; n = 6 control, 8 stress), GluA2 with PSD-95 (Figure 3B; t _(8)_ = 4.590, **p = 0.002; n = 5 control, 8 stress), and PKMζ with GluA2 (Figure 3C; t _(5)_ = 2.645, *p = 0.046; n = 6 control, 6 stress). No significant differences between overall levels were found between conditions for IP of PSD-95 (Figure 3D) or IP of GluA2 (Figure 3E). 

### Platform stress increased mature spines in CA1

The expression of spine number and type associated with various stress conditions can be indicative of short- and long-term effects of stress. Rapid stress effects on spines are associated with changes in spine density [12,18,42] while chronic effects of stress are associated with changes in dendritic morphology [43–45]. The mechanisms driving these modifications in spine morphology are known to involve a range of neurotransmitters, growth factors and hormones [46,47]. However, the underlying expression pattern of various synaptic markers within these changing spines in vivo is largely unknown and difficult to measure, except with gold-impregnated electron microscopy. Traditional Golgi-Cox staining, while useful for determining changes in spine density, has limited application in identifying spine shape. To circumvent these limitations, we utilized a new technique that allows for simultaneous immunohistochemistry and Golgi-Cox staining [33]. Recently, imaging of Golgi staining by confocal microscopy has been shown to provide enhanced 3D resolution of neuronal dendrites and spines beyond the visual resolution of traditional Golgi using brightfield microscopy alone [48]. Additional analysis of these images by customizable algorithms in IMARIS software allows for the reliable quantification of specific spine shapes [35]. The combination of Golgi-Cox staining with immunohistochemistry further enhances the spine analysis with the colocalization of various synaptic markers within specific spine types and allows for the identification of these changes within discrete hippocampal sub-regions. Within the CA1 subfield (Figure 4E), stress significantly increased long-thin (F_(1,20)_ = 6.774, *p = 0.017; n = 10 control dendrites, 12 stress) and mushroom (F_(1,21)_ = 8.567, **p = 0.008; n = 11 control, 12 stress) spine types. Stress produced a corresponding decrease in filopodia (F_(1,19)_ = 5.854, *p = 0.026; n = 10 control, 11 stress) and no change in stubby spines (F_(1,21)_ = 0.191, p = 0.666; n = 11 control, 12 stress). In long-thin spines (Figure 4H), there were increases in GluA2 (F_(1,18)_ = 9.006,**p = 0.008), PSD-95 (F_(1,18)_ = 10.252,**p = 0.005) and their colocalization (F_(1,18)_ = 10.280,**p = 0.005; n = 10 control, 10 stress). A similar effect was observed in mushroom spines (Figure 4I) with increases in GluA2 (F_(1,18)_ = 11.127, **p = 0.004), PSD-95 (F_(1,18)_ = 15.965, ***p = 0.0008) and in their colocalization (F_(1,18)_ = 8.266, *p = 0.010; n = 11 control, 9 stress). Stress did not alter the expression of GluA2, PSD-95 or their colocalization in filopodia and stubby spines (Figure 4F-G). Total spines counted were not significantly different between control and stress conditions (Table 2). 

**Table 2:** Total spine counts across hippocampal subfields.

	**CA1**		**CA3**		**Dentate gyrus**	
	**Control**	**Stress**	**Control**	**Stress**	**Control**	**Stress**
**Total spines**	531	529	749	447	920	1329
**Avg. spines per dendrite**	44.3	44.1	62.4	39.9	76.7	110.8
**SD**	14.1	9.3	23.8	20.8	11.0	18.4
**p**	0.987		0.285		0.064	
**Avg. dendrite length**	32.8	30.3	53.6	43.1	38.8	44.7
**SD**	4.9	2.0	7.6	7.3	9.1	10.7
**p**	0.489		0.160		0.505	

### Platform stress decreased immature spines in CA3

In contrast to what we observed in CA1, in CA3 (Figure 5E) stress had no effect on mushroom spine expression (F_(1,21)_ = 0.512, p = 0.482; n = 12 control dendrites, 11 stress), but decreased filopodia significantly (F_(1,18)_ = 6.911, *p = 0.017; n = 11 control, 9 stress). Stubby spines also demonstrated a trend towards decreased expression (F_(1,21)_ = 4.322, p = 0.050; n = 12 control, 11 stress). In addition, long-thin spines did significantly increase in this subfield (F_(1,20)_ = 4.388, *p = 0.049; n = 11 control, 11 stress). Filopodia (Figure 5F) showed a decrease in GluA2 (F_(1,15)_ = 5.332, *p = 0.036), PSD-95 (F_(1,15)_ = 6.340, *p = 0.024) and in their colocalization (F_(1,15)_ = 5.002, *p = 0.041; n = 9 control, 8 stress). Within stubby spines (Figure 5G), there were decreases in GluA2 (F_(1,15)_ = 5.836, *p = 0.029) and PSD-95 (F_(1,15)_ = 6.436, *p = 0.023) expression but no significant change in their colocalization (F_(1,15)_ = 4.342, p = 0.055; n = 9 control, 8 stress). No changes in GluA2, PSD-95 or their colocalization were found in long-thin spines (Figure 5H). Stress did increase the expression of GluA2 in mushroom spines (F_(1,19)_ = 7.710, *p = 0.012; n = 12 control, 9 stress) but did not change the expression of PSD-95 or their colocalization (Figure 5I). Total spines counted were not significantly different between control and stress conditions (Table 2).

### Platform stress increased both immature and mature spines in DG-OML

In an effort to further understand the changing spine density and synaptic marker expression induced in various hippocampal subfields after stress, we also focused on the outer molecular layer of the dentate gyrus. The OML is directly activated by discrete projections from the entorhinal cortex (EC), which itself is activated by terminals fibers originating from hippocampal CA1. The superficial layers of EC project almost exclusively to the OML [49], connecting activity in CA1 to OML through EC [50]. Following acute stress, spine counts (Figure 6E) for neither filopodia (F_(1,22)_ = 0.069, p = 0.796; n = 12 control dendrites, 12 stress) nor mushroom spines (F_(1,18)_ = 0.119, p = 0.734; n = 10 control, 10 stress) were affected by stress. However, we found that stress significantly increased stubby (F_(1,20)_ = 5.192, *p = 0.037; n = 11 control, 11 stress) and long-thin spines (F_(1,20)_ = 6.956, *p = 0.016; n = 12 control, 10 stress). The pattern of changing spine morphology for stubby and long-thin spines also demonstrated significant changes in the expression of synaptic markers. Stubby spines (Figure 6G) showed increases in GluA2 (F_(1,18)_ = 12.037, **p = 0.003), PSD-95 (F_(1,18)_ = 10.142, **p = 0.005) and their colocalization (F_(1,18)_ = 6.702, *p = 0.019; n = 10 control, 10 stress). Similarly, long-thin spines (Figure 6H) also showed increases in GluA2 (F_(1,22)_ = 12.092, **p = 0.002), PSD-95 (F_(1,22)_ = 23.469, ***p = 0.0001) and their colocalization (F_(1,22)_ = 8.027, *p = 0.010; n = 12 control, 12 stress). No changes were observed in synaptic marker expression for filopodia (Figure 6F) or mushroom spines (Figure 6I). Total spines counted were not significantly different between control and stress conditions (Table 2).

## Discussion

In this report, we aimed to examine the effects of an acute, physiological stressor on memory function and markers of synaptic plasticity. We began by characterizing the effects of 1-hour platform stress on memory retrieval and found that platform stress produced significant deficits in memory retrieval for both a short-term memory involving object placement and also a long-term memory involving radial arm maze (Figure 1). The stress-induced memory deficits on the RAM were restricted to reference memory, without any deficits in short-term working memory, indicating that platform stress selectively undermines long-term memory retrieval. Interestingly, we show that stress also produced changes in the individual expression of markers from the PSD-enriched fraction of the hippocampus, with a significant increase in synaptic PKMζ and a concomitant decrease in synaptic GluA2 (Figure 2B-C). At the same time, total levels of GluA2 precipitated from hippocampal homogenate did not change (Figure 3E). Taken together, these results suggest that stress induces GluA2 to move out of the synapse, with the subunit possibly being sequestered in extra-synaptic membrane or taken up by endocytosis. Previous studies examining AMPA receptor mobility in culture have shown that the stress hormone corticosterone enhances surface mobility of GluA2 without affecting total GluA2 levels [41,51]. Additional work implicating internalization of the receptor (reducing synaptic GluA2) with memory impairment [22,23,28,52] is consistent with our behavioral results (Figure 1).

While stress resulted in divergent effects on PKMζ and GluA2 as individual synaptic markers, their co-immunoprecipitation together with PSD-95 increased in the total homogenate (Figure 3). In conjuction with increased synaptic PKMζ levels (Figure 2), these results are consistent with what we know of PKMζ colocalization with both GluA2 and PSD-95. In separate experiments, overexpression of PKMζ in cultured neurons has been shown to increase colocalization of GluA2/PSD-95 and to promote spine maturation [19,29]. Additionally, induction of chemical LTP in culture has been shown to increase clustering of PKMζ and PSD-95 [19].

It is important to note that our results in Figures 2, 3 illustrate the effects of stress on the expression and interaction of these markers in whole hippocampi. As stress has been shown to affect hippocampal function and plasticity differently depending on sub-region [2–4,7], we examined the expression and localization of GluA2 and PSD-95 in different spine types within particular hippocampus sub-fields using combined Golgi-IHC. The Golgi-IHC data revealed discrete effects observed in areas CA1, CA3 and DG-OML. These differences are consistent with glucocorticoid receptor (GR) and mineralocorticoid receptor (MR) expression in these areas [3,4], which may play a significant role in each area’s vulnerability to stress and/or their role in creating a new, stress-induced memory. We hypothesize that mature spines are produced with stress in CA1 since this sub-region has a high expression of the low affinity GR [53], which upon activation increase protein synthesis and AMPAR insertion via exocytosis [41], thus, explaining increased localization of GluA2 in CA1 spines (Figure 4H-I). Conversely, in CA3 where MR receptors are highly expressed [53], opposite effects can be observed. MR activation by corticosterone induces the movement of GluA2 subunits away from the synapse (lateral diffusion) through non-genomic functions, which may mediate the decrease in GluA2 within spines in CA3 (Figure 5F-G). Within the dentate gyrus, the OML receives spatial information from entorhinal cortex (EC), which itself is activated by inputs from CA1 [54,55]. This suggests that the OML can reflect activity similar to that observed in CA1 via EC and may also be encoding the stress experience as a new memory with increases in stubby and long-thin spine formation. This effect is consistent with a report showing increased PKMζ/GluA2 colocalization within spines in the dentate gyrus of monkeys with better scores on the delayed-nonmatch to sample test [52]. 

Spines demonstrate dynamic changes in morphology, forming from filopodia that do not contain post-synaptic densities and have few AMPARs [18]. In particular, GluA2-containing AMPARs are necessary for spine formation and stabilization [56]. In addition, spines undergo continuous turnover and replacement, an activity that can be altered under various conditions including sensory input during development [57,58], memory [59], and stress [46,47]. The increases in GluA2, PSD-95 and their colocalization within mature spines in CA1 are consistent with the understanding that synaptic maturation is associated with increased stability and resistance to disassembly [60]. Changes in spine morphology provide a predictable measure for shifts in stability and synaptic strength; large spines form stronger, longer lasting synapses while small spines are generally transient, forming weaker synapses [13,15,17]. This current understanding fits with our interpretation of the changes that are occurring after stress. We observe that within CA1 the changing spine morphology moves towards an increase in mature spines, in particular mushroom spines, which have been hypothesized to represent physical substrates of long-term memories [17]. Inherent to spine stabilization is PSD-95, an abundant structural protein fundamental to the organization of the spine [61,62]. In most cases, we found that increases in PSD-95 were also matched by increases in GluA2 and their colocalization, which is consistent with what is observed during LTP [63,64]. We infer that these synaptic changes highlight the interaction of GluA2, PKMζ and PSD-95 in spine stabilization. Further experiments are needed to determine the exact mechanisms by which stress activates pathways important to AMPAR subunit trafficking and spine stabilization.

The dendritic changes reported here may in fact underlie both deficits in the retrieval of previously acquired memories and the formation of new memories associated with the stress experience (Figure 1). GR activation immediately after training promotes memory consolidation [65] but immediately before retention testing impairs memory retrieval [38,66]. The shift to more mature spines in CA1 concomitant with an increase in GluA2 and PSD-95 colocalization is consistent with consolidation of a new stress memory, as GluA2 expression also increases within mushroom spines after fear conditioning [67]. A reduction of immature spines in CA3 is consistent with the elimination of spines after stress [68] and diminished synaptic GluA2 is associated with non-genomic activation of MR [41]. The increase in synaptic PKMζ as a consequence of platform stress suggests that PKMζ may be actively creating a stress memory that is undermining or in conflict with previously encoded memories. Thus, differential shifts in spine morphology between hippocampal subfields may reflect mechanisms involved in enhancing and/or impairing different types of memory processing in parallel. CA3 appears important for the retrieval of short-term spatial and/or novel information [69–71]. When long-term retention is required, the involvement of CA3 is diminished and CA1 begins to play a larger role [72]. These reports suggest that the 1h platform experience may be sufficiently long to activate the long-term memory encoding mechanisms. It is interesting to speculate that these rapid changes in spine morphology and clustering of synaptic markers may reflect long-term memory consolidation of a stress memory. Future studies are needed to dissect out the exact timing and contributions of these shifts across hippocampal subfields and along the dorsoventral axis of the hippocamapus, which also plays a role in stress-induced memory impairments [73].

While much is known about the effects of stress on learning, much less is known about the effects of stress on memory maintenance and/or retrieval and their associated synaptic architecture. Our stress paradigm generated impairments in retrieval whether the memory was encoded only a few hours before or over a period of many days. These results may hint at the vulnerability of even life-long memories to stress during retrieval, at which point, they can be much more malleable [74]. The stress response, which re-sculpts the pattern of spines, may make memories vulnerable to disruption, in much the same way as re-consolidation [74]. Thus, the stress effects described here, involving changing spine morphology and synaptic clustering, may reflect basic mechanisms that are compromised in diseases of memory. In Alzheimer’s disease, for example, neurofibrillary tangles within the hippocampus, medial temporal cortex, and amygdala show high expression of PKMζ and GluA2 aggregates [75]. 

These data are the first demonstration of a potential molecular mechanism underlying stress-induced memory impairment involving changing spine types across various hippocampus subfields. The stress-induced changes in the expression and clustering of GluA2, PKMζ and PSD-95 can be seen immediately after the 1h stressor, which is the same time point at which the retention tests in Figure 1 were administered. Though these markers are typically associated with memory maintenance, it is possible that under stress conditions, their clustering may impair maintenance and/or retrieval processes for previously acquired memories. Here we use a single stressor, which equally impairs a recently acquired memory or a memory acquired over several days, to highlight the sensitivity of memory to stress and the need to further investigate overlapping mechanisms between the two. Examining memory maintenance and/or retrieval processes in a variety of contexts has wide implications for understanding differences in short- and long-term memory mechanisms and also dysfunction of memory involving PKMζ and GluA2.

## References

[1] Joëls M, Fernandez G, Roozendaal B (2011) Stress and emotional memory: a matter of timing. Trends Cogn Sci 15: 280-288. doi:10.1016/j.tics.2011.04.004. PubMed: 21571575. 21571575

[2] Leuner B, Shors TJ (2012) Stress, anxiety, and dendritic spines: What are the connections? Neuroscience, 251: 108–19. doi:10.1016/j.neuroscience.2012.04.021. PubMed: 22522470. 22522470

[3] Joëls M, Baram TZ (2009) The neuro-symphony of stress. Nat Rev Neurosci 10: 459-466. PubMed: 19339973. 19339973 10.1038/nrn2632 PMC2844123

[4] Maras PM, Baram TZ (2012) Sculpting the hippocampus from within: stress, spines, and CRH. Trends Neurosci 35: 315-324. doi:10.1016/j.tins.2012.01.005. PubMed: 22386641. 22386641 PMC3423222

[5] Gourley SL, Kedves AT, Olausson P, Taylor JR (2009) A history of corticosterone exposure regulates fear extinction and cortical NR2B, GluR2/3, and BDNF. Neuropsychopharmacology 34: 707-716. doi:10.1038/npp.2008.123. PubMed: 18719621. 18719621 PMC3679657

[6] Lupien SJ, McEwen BS, Gunnar MR, Heim C (2009) Effects of stress throughout the lifespan on the brain, behaviour and cognition. Nat Rev Neurosci 10: 434-445. doi:10.1038/nrn2639. PubMed: 19401723. 19401723

[7] Kim JJ, Diamond DM (2002) The stressed hippocampus, synaptic plasticity and lost memories. Nat Rev Neurosci 3: 453-462. doi:10.1038/nrm832. PubMed: 12042880. 12042880

[8] Magariños AM, McEwen BS (1995) Stress-induced atrophy of apical dendrites of hippocampal CA3c neurons: comparison of stressors. Neuroscience 69: 83-88. doi:10.1016/0306-4522(95)00256-I. PubMed: 8637635. 8637635

[9] Luine V, Villegas M, Martinez C, McEwen BS (1994) Repeated stress causes reversible impairments of spatial memory performance. Brain Res 639: 167-170. doi:10.1016/0006-8993(94)91778-7. PubMed: 8180832. 8180832

[10] Conrad CD, Galea LA, Kuroda Y, McEwen BS (1996) Chronic stress impairs rat spatial memory on the Y maze, and this effect is blocked by tianeptine pretreatment. Behav Neurosci 110: 1321-1334. doi:10.1037/0735-7044.110.6.1321. PubMed: 8986335. 8986335

[11] Sandi C, Davies HA, Cordero MI, Rodriguez JJ, Popov VI et al. (2003) Rapid reversal of stress induced loss of synapses in CA3 of rat hippocampus following water maze training. Eur J Neurosci 17: 2447-2456. doi:10.1046/j.1460-9568.2003.02675.x. PubMed: 12814376. 12814376

[12] Holtmaat A, Svoboda K (2009) Experience-dependent structural synaptic plasticity in the mammalian brain. Nat Rev Neurosci 10: 647-658. doi:10.1038/nrn2699. PubMed: 19693029. 19693029

[13] Matsuzaki M, Honkura N, Ellis-Davies GC, Kasai H (2004) Structural basis of long-term potentiation in single dendritic spines. Nature 429: 761-766. doi:10.1038/nature02617. PubMed: 15190253. 15190253 PMC4158816

[14] Zhou Q, Homma KJ, Poo MM (2004) Shrinkage of dendritic spines associated with long-term depression of hippocampal synapses. Neuron 44: 749-757. doi:10.1016/j.neuron.2004.11.011. PubMed: 15572107. 15572107

[15] Yasumatsu N, Matsuzaki M, Miyazaki T, Noguchi J, Kasai H (2008) Principles of long-term dynamics of dendritic spines. J Neurosci 28: 13592-13608. doi:10.1523/JNEUROSCI.0603-08.2008. PubMed: 19074033. 19074033 PMC2706274

[16] Hotulainen P, Hoogenraad CC (2010) Actin in dendritic spines: connecting dynamics to function. J Cell Biol 189: 619-629. doi:10.1083/jcb.201003008. PubMed: 20457765. 20457765 PMC2872912

[17] Kasai H, Matsuzaki M, Noguchi J, Yasumatsu N, Nakahara H (2003) Structure-stability-function relationships of dendritic spines. Trends Neurosci 26: 360-368. doi:10.1016/S0166-2236(03)00162-0. PubMed: 12850432. 12850432

[18] Bourne JN, Harris KM (2008) Balancing structure and function at hippocampal dendritic spines. Annu Rev Neurosci 31: 47-67. doi:10.1146/annurev.neuro.31.060407.125646. PubMed: 18284372. 18284372 PMC2561948

[19] Shao CY, Sondhi R, van de Nes PS, Sacktor TC (2012) PKMzeta is necessary and sufficient for synaptic clustering of PSD-95. Hippocampus 22: 1501-1507. doi:10.1002/hipo.20996. PubMed: 22378468. 22378468 PMC3371310

[20] Ling DS, Benardo LS, Serrano PA, Blace N, Kelly MT et al. (2002) Protein kinase Mzeta is necessary and sufficient for LTP maintenance. Nat Neurosci 5: 295-296. doi:10.1038/nn829. PubMed: 11914719. 11914719

[21] Serrano P, Yao Y, Sacktor TC (2005) Persistent phosphorylation by protein kinase Mzeta maintains late-phase long-term potentiation. J Neurosci 25: 1979-1984. doi:10.1523/JNEUROSCI.5132-04.2005. PubMed: 15728837. 15728837 PMC6726070

[22] Ling DS, Benardo LS, Sacktor TC (2006) Protein kinase Mzeta enhances excitatory transmission by increasing the number of active postsynaptic AMPA receptors. Hippocampus 16: 443-452. doi:10.1002/hipo.20171. PubMed: 16463388. 16463388

[23] Yao Y, Kelly MT, Sajikumar S, Serrano P, Tian D et al. (2008) PKM zeta maintains late long-term potentiation by N-ethylmaleimide-sensitive factor/GluR2-dependent trafficking of postsynaptic AMPA receptors. J Neurosci 28: 7820-7827. doi:10.1523/JNEUROSCI.0223-08.2008. PubMed: 18667614. 18667614 PMC2597488

[24] Pastalkova E, Serrano P, Pinkhasova D, Wallace E, Fenton AA et al. (2006) Storage of spatial information by the maintenance mechanism of LTP. Science 313: 1141-1144. doi:10.1126/science.1128657. PubMed: 16931766. 16931766

[25] Shema R, Sacktor TC, Dudai Y (2007) Rapid erasure of long-term memory associations in the cortex by an inhibitor of PKM zeta. Science 317: 951-953. doi:10.1126/science.1144334. PubMed: 17702943. 17702943

[26] Serrano P, Friedman EL, Kenney J, Taubenfeld SM, Zimmerman JM et al. (2008) PKMzeta maintains spatial, instrumental, and classically conditioned long-term memories. PLOS Biol 6: 2698-2706. PubMed: 19108606. 19108606 10.1371/journal.pbio.0060318 PMC2605920

[27] Madroñal N, Gruart A, Sacktor TC, Delgado-García JM (2010) PKMzeta inhibition reverses learning-induced increases in hippocampal synaptic strength and memory during trace eyeblink conditioning. PLOS ONE 5: e10400. doi:10.1371/journal.pone.0010400. PubMed: 20454458. 20454458 PMC2861600

[28] Migues PV, Hardt O, Wu DC, Gamache K, Sacktor TC et al. (2010) PKMzeta maintains memories by regulating GluR2-dependent AMPA receptor trafficking. Nat Neurosci 13: 630-634. doi:10.1038/nn.2531. PubMed: 20383136. 20383136

[29] Ron S, Dudai Y, Segal M (2012) Overexpression of PKMζ alters morphology and function of dendritic spines in cultured cortical neurons. Cereb Cortex 22: 2519-2528. doi:10.1093/cercor/bhr323. PubMed: 22123937. 22123937 PMC4705334

[30] Schrott LM, Franklin L', Serrano PA (2008) Prenatal opiate exposure impairs radial arm maze performance and reduces levels of BDNF precursor following training. Brain Res 1198: 132-140. doi:10.1016/j.brainres.2008.01.020. PubMed: 18262500. 18262500 PMC2696491

[31] Sacktor TC, Osten P, Valsamis H, Jiang X, Naik MU et al. (1993) Persistent activation of the zeta isoform of protein kinase C in the maintenance of long-term potentiation. Proc Natl Acad Sci U S A 90: 8342-8346. doi:10.1073/pnas.90.18.8342. PubMed: 8378304. 8378304 PMC47352

[32] Noguès X, Micheau J, Jaffard R (1994) Protein kinase C activity in the hippocampus following spatial learning tasks in mice. Hippocampus 4: 71-77. doi:10.1002/hipo.450040109. PubMed: 8061753. 8061753

[33] Spiga S, Acquas E, Puddu MC, Mulas G, Lintas A et al. (2011) Simultaneous Golgi-Cox and immunofluorescence using confocal microscopy. Brain Struct Funct 216: 171-182. doi:10.1007/s00429-011-0312-2. PubMed: 21461741. 21461741 PMC3155021

[34] Harris KM, Jensen FE, Tsao B (1992) Three-dimensional structure of dendritic spines and synapses in rat hippocampus (CA1) at postnatal day 15 and adult ages: implications for the maturation of synaptic physiology and long-term potentiation. J Neurosci 12: 2685-2705. PubMed: 1613552. 1613552 10.1523/JNEUROSCI.12-07-02685.1992 PMC6575840

[35] Shen HW, Toda S, Moussawi K, Bouknight A, Zahm DS et al. (2009) Altered dendritic spine plasticity in cocaine-withdrawn rats. J Neurosci 29: 2876-2884. doi:10.1523/JNEUROSCI.5638-08.2009. PubMed: 19261883. 19261883 PMC2698814

[36] Nakagawa S, Hauber ME (2011) Great challenges with few subjects: statistical strategies for neuroscientists. Neurosci Biobehav Rev 35: 462-473. doi:10.1016/j.neubiorev.2010.06.003. PubMed: 20600287. 20600287

[37] Diamond DM, Fleshner M, Ingersoll N, Rose GM (1996) Psychological stress impairs spatial working memory: relevance to electrophysiological studies of hippocampal function. Behav Neurosci 110: 661-672. doi:10.1037/0735-7044.110.4.661. PubMed: 8864259. 8864259

[38] de Quervain DJ, Roozendaal B, McGaugh JL (1998) Stress and glucocorticoids impair retrieval of long-term spatial memory. Nature 394: 787-790. doi:10.1038/29542. PubMed: 9723618. 9723618

[39] Cazakoff BN, Johnson KJ, Howland JG (2010) Converging effects of acute stress on spatial and recognition memory in rodents: a review of recent behavioural and pharmacological findings. Prog Neuropsychopharmacol Biol Psychiatry 34: 733-741. doi:10.1016/j.pnpbp.2010.04.002. PubMed: 20394792. 20394792

[40] Hardt O, Migues PV, Hastings M, Wong J, Nader K (2010) PKMzeta maintains 1-day- and 6-day-old long-term object location but not object identity memory in dorsal hippocampus. Hippocampus 20: 691-695. PubMed: 19806657. 19806657 10.1002/hipo.20708

[41] Groc L, Choquet D, Chaouloff F (2008) The stress hormone corticosterone conditions AMPAR surface trafficking and synaptic potentiation. Nat Neurosci 11: 868-870. doi:10.1038/nn.2150. PubMed: 18622402. 18622402

[42] Kasai H, Fukuda M, Watanabe S, Hayashi-Takagi A, Noguchi J (2010) Structural dynamics of dendritic spines in memory and cognition. Trends Neurosci 33: 121-129. doi:10.1016/j.tins.2010.01.001. PubMed: 20138375. 20138375

[43] Pawlak R, Rao BS, Melchor JP, Chattarji S, McEwen B et al. (2005) Tissue plasminogen activator and plasminogen mediate stress-induced decline of neuronal and cognitive functions in the mouse hippocampus. Proc Natl Acad Sci U S A 102: 18201-18206. doi:10.1073/pnas.0509232102. PubMed: 16330749. 16330749 PMC1312427

[44] Diamond DM, Campbell AM, Park CR, Woodson JC, Conrad CD et al. (2006) Influence of predator stress on the consolidation versus retrieval of long-term spatial memory and hippocampal spinogenesis. Hippocampus 16(7): 571-576. doi:10.1002/hipo.20188. PubMed: 16741974. 16741974

[45] Chen Y, Dubé CM, Rice CJ, Baram TZ (2008) Rapid loss of dendritic spines after stress involves derangement of spine dynamics by corticotropin-releasing hormone. J Neurosci 28: 2903-2911. doi:10.1523/JNEUROSCI.0225-08.2008. PubMed: 18337421. 18337421 PMC2409370

[46] Calabrese B, Halpain S (2005) Essential role for the PKC target MARCKS in maintaining dendritic spine morphology. Neuron 48: 77-90. doi:10.1016/j.neuron.2005.08.027. PubMed: 16202710. 16202710

[47] Segal M (2010) Dendritic spines, synaptic plasticity and neuronal survival: activity shapes dendritic spines to enhance neuronal viability. Eur J Neurosci 31: 2178-2184. doi:10.1111/j.1460-9568.2010.07270.x. PubMed: 20550565. 20550565

[48] Pinto L, Mateus-Pinheiro A, Morais M, Bessa JM, Sousa N (2012) Immuno-Golgi as a tool for analyzing neuronal 3D-dendritic structure in phenotypically characterized neurons. PLOS ONE 7: e33114. doi:10.1371/journal.pone.0033114. PubMed: 22427964. 22427964 PMC3299750

[49] Deller T, Martinez A, Nitsch R, Frotscher M (1996) A novel entorhinal projection to the rat dentate gyrus: direct innervation of proximal dendrites and cell bodies of granule cells and GABAergic neurons. J Neurosci 16: 3322-3333. PubMed: 8627369. 8627369 10.1523/JNEUROSCI.16-10-03322.1996 PMC6579155

[50] Witter MP (1993) Organization of the entorhinal-hippocampal system: a review of current anatomical data. Hippocampus 3: 33–44 Spec No: 33-44 PubMed: 8287110. 8287110

[51] Martin S, Henley JM, Holman D, Zhou M, Wiegert O et al. (2009) Corticosterone alters AMPAR mobility and facilitates bidirectional synaptic plasticity. PLOS ONE 4: e4714. doi:10.1371/journal.pone.0004714. PubMed: 19305644. 19305644 PMC2659165

[52] Hara Y, Punsoni M, Yuk F, Park CS, Janssen WG et al. (2012) Synaptic distribution of GluA2 and PKMζ in the monkey dentate gyrus and their relationships with aging and memory. J Neurosci 32: 7336-7344. doi:10.1523/JNEUROSCI.0605-12.2012. PubMed: 22623679. 22623679 PMC3391702

[53] Van Eekelen JA, De Kloet ER (1992) Co-localization of brain corticosteroid receptors in the rat hippocampus. Prog Histochem Cytochem 26: 250-258. doi:10.1016/S0079-6336(11)80102-6. PubMed: 1336613. 1336613

[54] McNaughton BL (1980) Evidence for two physiologically distinct perforant pathways to the fascia dentata. Brain Res 199: 1-19. doi:10.1016/0006-8993(80)90226-7. PubMed: 7407615. 7407615

[55] Hargreaves EL, Rao G, Lee I, Knierim JJ (2005) Major dissociation between medial and lateral entorhinal input to dorsal hippocampus. Science 308: 1792-1794. doi:10.1126/science.1110449. PubMed: 15961670. 15961670

[56] Passafaro M, Nakagawa T, Sala C, Sheng M (2003) Induction of dendritic spines by an extracellular domain of AMPA receptor subunit GluR2. Nature 424: 677-681. doi:10.1038/nature01781. PubMed: 12904794. 12904794

[57] Holtmaat AJ, Trachtenberg JT, Wilbrecht L, Shepherd GM, Zhang X et al. (2005) Transient and persistent dendritic spines in the neocortex in vivo. Neuron 45: 279-291. doi:10.1016/j.neuron.2005.01.003. PubMed: 15664179. 15664179

[58] Zuo Y, Lin A, Chang P, Gan WB (2005) Development of long-term dendritic spine stability in diverse regions of cerebral cortex. Neuron 46: 181-189. doi:10.1016/j.neuron.2005.04.001. PubMed: 15848798. 15848798

[59] Kandel ER (2001) The molecular biology of memory storage: a dialogue between genes and synapses. Science 294: 1030-1038. doi:10.1126/science.1067020. PubMed: 11691980. 11691980

[60] Garner CC, Waites CL, Ziv NE (2006) Synapse development: still looking for the forest, still lost in the trees. Cell Tissue Res 326: 249-262. doi:10.1007/s00441-006-0278-1. PubMed: 16909256. 16909256

[61] Sheng M, Hoogenraad CC (2007) The postsynaptic architecture of excitatory synapses: a more quantitative view. Annu Rev Biochem 76: 823-847. doi:10.1146/annurev.biochem.76.060805.160029. PubMed: 17243894. 17243894

[62] Blanpied TA, Kerr JM, Ehlers MD (2008) Structural plasticity with preserved topology in the postsynaptic protein network. Proc Natl Acad Sci U S A 105: 12587-12592. doi:10.1073/pnas.0711669105. PubMed: 18723686. 18723686 PMC2519044

[63] El-Husseini Ael D, Schnell E, Dakoji S, Sweeney N, Zhou Q et al. (2002) Synaptic strength regulated by palmitate cycling on PSD-95. Cell 108: 849-863. doi:10.1016/S0092-8674(02)00683-9. PubMed: 11955437. 11955437

[64] Ehrlich I, Malinow R (2004) Postsynaptic density 95 controls AMPA receptor incorporation during long-term potentiation and experience-driven synaptic plasticity. J Neurosci 24: 916-927. doi:10.1523/JNEUROSCI.4733-03.2004. PubMed: 14749436. 14749436 PMC6729816

[65] Roozendaal B, Hernandez A, Cabrera SM, Hagewoud R, Malvaez M et al. (2010) Membrane-associated glucocorticoid activity is necessary for modulation of long-term memory via chromatin modification. J Neurosci 30: 5037-5046. doi:10.1523/JNEUROSCI.5717-09.2010. PubMed: 20371824. 20371824 PMC2861482

[66] Roozendaal B (2002) Stress and memory: opposing effects of glucocorticoids on memory consolidation and memory retrieval. Neurobiol Learn Mem 78: 578-595. doi:10.1006/nlme.2002.4080. PubMed: 12559837. 12559837

[67] Matsuo N, Reijmers L, Mayford M (2008) Spine-type-specific recruitment of newly synthesized AMPA receptors with learning. Science 319: 1104-1107. doi:10.1126/science.1149967. PubMed: 18292343. 18292343 PMC2692967

[68] Kole MH, Costoli T, Koolhaas JM, Fuchs E (2004) Bidirectional shift in the cornu ammonis 3 pyramidal dendritic organization following brief stress. Neuroscience 125: 337-347. doi:10.1016/j.neuroscience.2004.02.014. PubMed: 15062977. 15062977

[69] Kesner RP, Rolls ET (2001) Role of long-term synaptic modification in short-term memory. Hippocampus 11: 240-250. doi:10.1002/hipo.1040. PubMed: 11769307. 11769307

[70] Kesner RP, Hunsaker MR, Warthen MW (2008) The CA3 subregion of the hippocampus is critical for episodic memory processing by means of relational encoding in rats. Behav Neurosci 122: 1217-1225. doi:10.1037/a0013592. PubMed: 19045941. 19045941

[71] Kesner RP (2013) A process analysis of the CA3 subregion of the hippocampus. Front Cell Neurosci 122: 1217-1225. 10.3389/fncel.2013.00078 PMC3664330 23750126

[72] Lee I, Kesner RP (2003) Differential roles of dorsal hippocampal subregions in spatial working memory with short versus intermediate delay. Behav Neurosci 117: 1044-1053. doi:10.1037/0735-7044.117.5.1044. PubMed: 14570553. 14570553

[73] Dorey R, Piérard C, Chauveau F, David V, Béracochéa D (2012) Stress-induced memory retrieval impairments: different time-course involvement of corticosterone and glucocorticoid receptors in dorsal and ventral hippocampus. Neuropsychopharmacology 37: 2870-2880. doi:10.1038/npp.2012.170. PubMed: 22948976. 22948976 PMC3499833

[74] Finnie PS, Nader K (2012) The role of metaplasticity mechanisms in regulating memory destabilization and reconsolidation. Neurosci Biobehav Rev 36: 1667-1707. doi:10.1016/j.neubiorev.2012.03.008. PubMed: 22484475. 22484475

[75] Crary JF, Shao CY, Mirra SS, Hernandez AI, Sacktor TC (2006) Atypical protein kinase C in neurodegenerative disease I: PKMzeta aggregates with limbic neurofibrillary tangles and AMPA receptors in Alzheimer disease. J Neuropathol Exp Neurol 65: 319-326. doi:10.1097/01.jnen.0000218442.07664.04. PubMed: 16691113. 16691113

